# Enhancement of Lipid Stability of Frozen Fish by Octopus-Waste Glazing

**DOI:** 10.3390/foods12122298

**Published:** 2023-06-07

**Authors:** Lucía Méndez, Bin Zhang, Santiago P. Aubourg

**Affiliations:** 1 Department of Food Technology, Marine Research Institute (CSIC), c/Eduardo Cabello, 6, 36208 Vigo, Spain; luciamendez@iim.csic.es; 2 Key Laboratory of Health Risk Factors for Seafood of Zhejiang Province, College of Food Science and Pharmacy, Zhejiang Ocean University, Zhoushan 316022, China; zhangbin@zjou.edu.cn

**Keywords:** horse mackerel, octopus-cooking liquor, oxidation, hydrolysis, ω3 fatty acids, rancidity, quality

## Abstract

The antioxidant properties of the liquor resulting from commercial octopus cooking were analysed for this study. Two different concentrations of octopus-cooking liquor (OCL) were tested as glazing systems during the frozen storage period (−18 °C for up to 6 months) of whole Atlantic horse mackerel (*Trachurus trachurus*). Compared to water-control glazing samples, an inhibitory effect (*p* < 0.05) on lipid oxidation development (the formation of thiobarbituric acid reactive substances and fluorescent compounds) was detected in frozen fish treated with the most concentrated OCL-glazing system. Additionally, a preservative effect (*p* < 0.05) on polyunsaturated fatty acids (measurement of polyene index) was also proved. However, no effect (*p* > 0.05) on the free fatty acid content and on the ω3/ω6 ratio was detected with the presence of the OCL in the glazing system. An increased lipid quality in frozen horse mackerel was established by including the OCL solution in the glazing system. According to previous research, the observed preserving properties were explained on the basis of the presence of antioxidant compounds in the cooking liquor. A novel and valuable combination of glazing processing and the employment of a marine waste substrate is proposed to enhance the lipid stability of frozen fish.

## 1. Introduction

Although frozen storage can inhibit microbial spoilage, seafood constituents may undergo deteriorative modifications such as protein insolubility, aggregate formation, mechanical damage, and lipid hydrolysis and oxidation [1,2,3]. Therefore, different complementary strategies have been applied to extend the shelf-life time of frozen seafood. One particular technology is the use of an ice layer on the surface of a frozen product, which is referred to as glazing [4,5]. Thus, the appropriate glazing of seafood prior to the freezing period would protect the final product from dehydration, quality loss, and oxidation. Previous studies have shown the valuable effects of glazing by inhibiting lipid oxidation [6,7] and hydrolysis [8,9] development, volatile amine formation [10,11], and microbial activity [12,13]. Additionally, the glazing process has been shown to improve the pH value and water-holding capacity, colour, and textural properties of frozen fish [10,11].

Seafood processing is considered to be one of the main sources of fishing by-products, since only 50–60% of the total catch is employed for human consumption [14,15]. Hence, large volumes of undesired products are obtained; as a result, marine waste constitutes an important source of environmental contamination, unless recovery efforts are carried out and the commercial value of the waste can be increased [16,17]. The most common marine by-products include fish oil and meal, which provide an interesting source of high-value lipids and proteins, respectively. However, recent reports have shown that seafood by-products also contain healthy and valuable components such as vitamins, chitin, enzymes, amino acids, minerals, pigments, and collagen [18,19].

Among cephalopods, octopus species represent highly nutritional seafood that can be commercialised in a wide range of products. Octopus processing generates a wide range of by-products, which include remarkable functional values that have promising potential for the integrated exploitation and employment of bioactive molecules [20,21]. As an octopus processing waste product, cooking juice or liquor has attracted the interest of food technologists. Thus, Oh et al. [22] observed remarkable antioxidant and antihypertensive effects in cooking juices resulting from skipjack tuna, oyster, and octopus processing, while Kim et al. [23] showed that cooking drip obtained from Giant Pacific octopus (*Enteroctopus dofleini*) processing had a radical scavenging activity and an inhibitory activity against tyrosine and angiotensin I converting enzyme. Recently, the addition of octopus (*Octopus vulgaris*) cooking juice as a coating medium led to an increased lipid quality in water-canned Chub mackerel (*Scomber colias*) [24].

The current research focused on the lipid stability of fish during frozen storage. Its aim was to study the effect of glazing treatments, including an aqueous solution of octopus-cooking liquor (OCL) during the frozen storage of Atlantic horse mackerel (*Trachurus trachurus*). Two different concentrations of OCL were checked and compared to a control glazing (water-glazing) treatment. Quality changes of the lipid fraction were monitored after 3 and 6 months of frozen storage at −18 °C by the assessment of the lipid damage (oxidation and hydrolysis) indices and fatty acid (FA) analysis.

## 2. Materials and Methods

### 2.1. Initial OCL and Glazing Systems

Commercial OCL was prepared and provided by Frigoríficos Rosa de los Vientos S. L. (Marín, Pontevedra, Spain). Cooking liquor resulting from common octopus (*Octopus vulgaris*) processing was filtered and kept in vacuum-sealed bottles protected from light. Liquor was kept under refrigerated conditions (4 °C) before use, according to commercial recommendations provided by the manufacturer.

Two glazing systems were prepared including the OCL. Aq. 10% and 30% solutions of OCL were prepared in order to obtain low- and high-concentrated glazing systems (OCL-10 and OCL-30, respectively). A control glazing system was prepared with water and without the presence of the OCL (Control condition).

Concentrations of OCL employed in the current study were chosen according to various preliminary studies. Thus, aqueous solutions including OCL in the 5–100% range were tested. As a result, concentrations higher than 30% were shown to modify some sensory descriptors (i.e., taste, colour, and odour) of frozen horse mackerel. Therefore, this concentration (30%) was taken into account in the present study, together with a less concentrated one (10%), to study the possible effect of the OCL concentration on the lipid quality of the frozen fish.

### 2.2. Initial Fresh Horse Mackerel, Freezing, Glazing, and Frozen Storage

Fresh horse mackerel (63 individuals) were obtained at the Vigo harbour (Galicia, Spain) and transported on ice to the laboratory. The weight and length of the individual fish ranged from 110 to 135 g and from 25.0 to 28.5 cm, respectively.

Once in the laboratory, nine individuals were separated, and considered as initial fresh fish. These individuals were divided into three different groups (three individuals per group) that were analysed independently to achieve the statistical analysis (*n* = 3). The remaining fish were divided into three batches (18 individuals in each batch) that were immediately frozen at −40 °C.

After the freezing step (−40 °C for 48 h), the three batches were immersed in the above-mentioned OCL-30, OCL-10, and Control glazing systems. In all batches, individuals were immersed for 30 s at 4 °C, allowed to drain for 15 s, packed in polyethylene bags (three individuals per bag) and stored at −18 °C.

Sampling was carried out at months 3 and 6 of storage at −18 °C. At each time and for each glazing condition, nine individuals were considered that were divided into three groups (three individuals per group) and considered separately (*n* = 3). Frozen fish were analysed after thawing, which was carried out with overnight storage in a cool room at 4 °C.

### 2.3. Proximate Analysis of OCL

The standard methods of the Association of Official Analytical Chemists (AOAC) were used to quantify the moisture, ash, lipid, and protein content in the OCL [25]. Results were calculated as g kg^−1^.

### 2.4. Lipid Extraction of Fish Muscle

The lipid fraction of the fish white muscle was analysed by using the Bligh and Dyer [26] method. This method employs a chloroform–methanol (1:1) mixture. The quantification of lipid extracts was carried out following the methodology proposed by Herbes and Allen [27]. The lipid content was determined as g kg^−1^ horse mackerel muscle.

### 2.5. Assessment of Lipid Damage of Fish Muscle

The peroxide value (PV) was determined by spectrophotometric (520 nm) (Beckman Coulter DU 640 spectrophotometer, Beckman Coulter Inc., Brea, CA, USA) analysis of the lipid extract by peroxide reduction with ferric thiocyanate [28]. The results were determined as meq. active oxygen kg^−1^ lipids.

The thiobarbituric acid (TBA) index was measured according to Vyncke [29]. The content of thiobarbituric acid reactive substances (TBARS) was spectrophotometrically determined (532 nm) and calculated from a standard curve employing 1,1,3,3-tetraethoxy-propane. The results were calculated as mg malondialdehyde kg^−1^ muscle.

The formation of fluorescent compounds (Fluorimeter LS 45; PerkinElmer España; Tres Cantos, Madrid, Spain) was measured in the lipid extract of the fish muscle according to previous research [30]. The relative fluorescence (RF) was calculated as follows: RF = *F/F_st_*, where *F* is the fluorescence measured at each excitation/emission wavelength pair (i.e., 393/463 and 327/415 nm) and *F_st_* is the fluorescence intensity of a quinine sulphate solution (1 µg mL^−1^ in 0.05 M H_2_SO_4_) at the corresponding wavelength pair. The results were determined as the fluorescence ration (FR), which was calculated as the ratio between the two RF values: FR = RF_393/463nm_/RF_327/415nm_.

The free fatty acid (FFA) value was determined in the lipid extract of the fish muscle according to the Lowry and Tinsley [31] method; this method is based on the complex formation of cupric acetate-pyridine, followed by spectrophotometric assessment at 715 nm. The results were calculated as g FFA kg^−1^ fish muscle.

### 2.6. FA Analysis of Fish Muscle

Lipid fractions were converted into FA methyl esters (FAME) by employing acetyl chloride in methanol and then analysed in an 8700 gas chromatograph (PerkinElmer España), equipped with a fused silica capillary column SP-2330 (0.25 mm i.d. × 30 m, 0.20 μm film, Supelco Inc., Bellefonte, PA, USA) [32]. Commercial standard mixtures (Qualmix Fish, Larodan, Malmo, Sweden; FAME mix, Supelco, Inc.) were employed for the identification of peaks corresponding to FAME. For quantitative purposes, peak areas were automatically integrated; C19:0 FA was used as internal standard.

The content of each FA was determined as g 100 g^−1^ total FA. Such values were used to calculate the content of FA groups (saturated FA, STFA; monounsaturated FA, MUFA; polyunsaturated FA, PUFA; total ω3 FA; and total ω6 FA) (g 100 g^−1^ total FA) and ratios (polyene index, PI; total ω3/total ω6). The PI was determined according to the following FA content ratio: (C20:5ω3 + C22:6ω3)/C16:0.

### 2.7. Statistical Analysis

Results obtained were subjected to the ANOVA method, in order to explore the differences resulting from the effect of the glazing condition and the frozen storage time. Three replicates (*n* = 3) were considered in the present research. The least-squares difference (LSD) method was employed to carry out the comparison of means. Analyses were performed using the PASW Statistics 18 software for Windows (SPSS Inc., Chicago, IL, USA). Differences were considered significant for a confidence interval at the 95-% level (*p* < 0.05). 

## 3. Results and Discussion

### 3.1. Determination of the Proximate Composition of OCL

The contents obtained for moisture, total proteins, total lipids and ash were (g kg^−1^) 972.0 ± 7.1, 8.2 ± 0.5, 1.9 ± 0.3, and 10.4 ± 0.6, respectively. The values obtained for moisture were higher than those reported for cooking liquor obtained from skipjack tuna (*Katsuwonus pelamis*) [33] and octopus, oyster, and skipjack tuna (*K. pelamis*) [22]. Contrarily, the current contents found for total proteins, total lipids, and ash were lower than those found in the mentioned studies [22,33]. 

### 3.2. Determination of the Lipid Oxidation Development of Fish Muscle

The development of lipid oxidation was measured by the analysis of primary (peroxides), secondary (TBARS), and tertiary (fluorescent compounds) compound formation.

The peroxide content was found to be low in all types of samples [2,3]; all values were included in the 0.46–1.51 meq. oxygen kg^−1^ lipids range (Table 1). For both the control and treated samples, the highest average values were detected after 3 months of frozen storage. In the case of the OCL-30 batch, this value was significantly higher (*p* < 0.05) than those obtained in their corresponding samples of the initial and 6-month period. No differences (*p* > 0.05) in the peroxide content were detected between Control samples and fish subjected to either of the two glazing treatments including the OCL.

A progressive formation of TBARS was detected in all batches as a result of the frozen storage time (Table 1). A significant increase (*p* < 0.05) with the frozen storage time was observed in fish subjected to the Control and OCL-30 glazing systems. After 3 and 6 months, a lower TBARS content (*p* < 0.05) was detected in fish corresponding to the highest OCL concentration than in their counterpart Control samples. No inhibitory effect (*p* > 0.05) was detected on the TBA value as a result of the glazing treatment including the lowest OCL concentration.

A comparison between initial fresh fish and frozen fish stored for 3 months revealed a remarkable formation (*p* < 0.05) of fluorescent compounds in all batches (Table 1). A general increase of average values was also observed after a 6-month period. An important inhibitory effect on the formation of fluorescent compounds was proved as a result of the glazing treatment. Thus, samples corresponding to OCL-treated conditions led to lower (*p* < 0.05) FR values when compared to the Control batch. No differences (*p* > 0.05) in the fluorescent compound content were detected between samples corresponding to both OCL-glazing batches.

Fish processing including freezing, frozen storage, and thawing has been reported to lead to a remarkable lipid oxidation development [30,31,34]. Such deterioration may be facilitated by lysis of mitochondria and lysosomes that alter the distribution of prooxidant enzymes (i.e., peroxidases, lipoxygenases, etc.) and factors affecting the rate of enzyme reactions in tissues, so that the oxidation damage in the frozen fish could be accelerated. Consequently, many different compounds are produced, most of them unstable and thus break down into compounds with a smaller molecular weight or that react with other compounds present in the fish muscle [2,3,35].

However, according to the results obtained in the present study, an inhibitory effect on the formation of secondary (i.e., TBARS) and tertiary (FR) oxidation compounds has been proved in frozen fish corresponding to the glazing batch with the highest OCL concentration. Therefore, an increased rancidity stability of the current fish species was identified during the freezing period.

The antioxidant properties of the OCL obtained from the current octopus species were already proved in seafood systems. Thus, an inhibitory effect on the formation of fluorescent compounds was detected in water-canned Chub mackerel (*S. colias*) when including OCL in the coating medium [24]. The employment of the OCL also provoked an inhibitory effect on lipid oxidation development (the formation of TBARS and fluorescent compounds) when including it in the coating medium of canned horse mackerel (*T. trachurus*) previously subjected to different frozen storage periods [36].

The antioxidant properties of cooking liquors obtained from several marine species have already been detected in different in vitro assays. Concerning octopus species, Oh et al. [22] studied the composition of the juice resulting from octopus cooking and proved an antioxidant behaviour (Rancimat assay). Based on the levels found for total protein and total extractive-N contents, this cooking drip was recommended to be employed as a source of functional seasoning. Similarly, the cooking juice obtained from Giant Pacific octopus (*E. dofleini*) revealed an antioxidant capacity (DPPH assay), this effect increasing with electron beam [23] or gamma [37] irradiation. In both studies, it was reasoned that the antioxidant activity was increased by structural modification of proteins in the cooking drip by the irradiation applied.

Abundant information concerning the antioxidant behaviour of cooking drips obtained from tuna species has been reported. After the enzymatic hydrolysis of tuna cooking juice, Jao and Ko [38] isolated seven peptides with antioxidant properties (i.e., DPPH radical assay); the peptide sequences comprised four to eight amino acid residues, which included Val, Ser, Pro, His, Ala, Asp, Lys, Glu, Gly, and Tyr. Cheong et al. [39] analysed the peptides obtained from skipjack tuna (*K. pelamis*) boiled extracts; the study demonstrated that low molecular weight peptides containing histidine (i.e., carnosine and anserine) were capable of inhibiting lipid oxidation (free radical scavengers and reducing agents). Additionally, recent research investigated the production of antioxidant peptides from tuna (*Thunnus albacares*) heads [40] and tuna steamed juice [41].

In agreement with the current study, previous related research accounts for lipid oxidation inhibition (peroxide and TBARS formation) as a result of applying a glazing system that includes different kinds of natural preservative compounds obtained from plant compounds. Thus, essential oil (sage, thyme or clove) extracts were employed with frozen rainbow trout (*Oncorhynchus mykiss*) fillets [8], green tea and grape seed extracts with frozen bonito (*Sarda sarda*) [5], and saponin-free quinoa (*Chenopodium quinoa*) extracts with frozen mackerel (*Scomber scombrus*) [42]. Additionally, natural antioxidant products obtained from seafood processing have also been added to glazing systems and have led to a quality enhancement. Thus, herring (*Clupea harengus*) muscle press juice was applied to frozen herring (*C. harengus*) fillets [43], a jumbo squid (*Dosidicus gigas*) skin extract to frozen mackerel (*S. colias*) [44], and an alga *Cystoseira stricta* extract to frozen mackerel (*S. colias*) [32]. An inhibitory effect on secondary lipid oxidation was detected by Wang and Xie [11] in frozen horse mackerel (*Trachurus japonicus*) by employing a theaflavin-glazing system; this effect was increased by employing a double-glazing treatment. A slight decrease of peroxide and TOTOX values was detected by Naseri et al. [10] in frozen rainbow trout (*O. mykiss*) by cold-water (1.0 °C for 20 s) glazing. An inhibitory effect on peroxide formation was detected in frozen shrimp (*Solenocera melantho*) by employing a glazing system that includes a rosemary (*Rosmarinus officinalis*) extract [9]. Recently, an inhibitory effect on the formation of secondary lipid oxidation compounds was detected in frozen squid by employing sodium erythorbate and sodium polyacrylate glazing [6], as well as in frozen big eye tuna (*Thunnus obesus*) by using glazing with rosmarinic acid [7].

### 3.3. Determination of the FA Composition of Fish Muscle

The analysis of the FA profile of the lipid extract obtained from the initial fresh fish revealed the following individual FA composition (g 100 g^−1^ total FA): 4.30 ± 0.15 (C14:0), 0.39 ± 0.02 (C15:0), 22.18 ± 0.83 (C16:0), 4.45 ± 0.01 (C16:1ω7), 1.22 ± 0.06 (C17:0), 7.54 ± 0.12 (C18:0), 16.08 ± 0.72 (C18:1ω9), 3.06 ± 0.33 (C18:1ω7), 1.47 ± 0.18 (C18:2ω6), 1.71 ± 0.09 (C20:1ω9), 0.33 ± 0.01 (C20:2ω6), 1.45 ± 0.18 (C20:4ω6), 0.25 ± 0.07 (C22:1ω9), 7.69 ± 0.28 (C20:5ω3), 0.43 ± 0.02 (C22:4ω6), 0.75 ± 0.03 (C24:1ω9), 2.88 ± 0.14 (C22:5ω3), and 23.84 ± 0.69 (C22:6ω3). FA analysis was carried out in all frozen samples. To better focus on possible FA changes, discussion of the FA results in the current research will be focused on the values obtained in the FA groups (STFA, MUFA, PUFA, and total ω3 FA) and ratios (polyene index, PI; total ω3/total ω6).

The STFA group showed values included in the 28.52–36.06 g 100 g^−1^ total FA range (Table 2). Samples corresponding to the Control batch revealed an increasing value (*p* < 0.05) with the storage time. Contrarily, frozen fish subjected to the glazing system including the highest OCL concentration showed a decrease (*p* < 0.05) in value with the storage time. A comparison of the batches revealed higher average levels in samples corresponding to the Control condition. After a 6-month period, lower values (*p* < 0.05) were observed in fish corresponding to the OCL-30 condition when compared to their counterparts from Control and OCL-10 batches.

In all batches under study, a lower level of MUFA than of STFA was detected (8.99–21.46 g 100 g^−1^ total FA range) (Table 2). In all cases, a decreasing content was observed with frozen storage time, this decrease being notably strong (*p* < 0.05) after 6 months of frozen storage. A comparison of the batches did not provide significant differences (*p* > 0.05), so that a definite trend of MUFA content resulting from the glazing treatment could not be proved.

The PUFA group showed to be more abundant than the STFA and MUFA groups in all cases (Table 2). The PUFA level showed a marked increase (*p* < 0.05) in all batches after 6 months of frozen storage. A comparison of the batches revealed higher average values in the frozen fish subjected to the glazing system including the highest OCL concentration. This difference was found to be significant (*p* < 0.05) after a 6-month period.

Remarkable total ω3 values were obtained in all batches; all values were included in the 41.98–58.70 g 100 g^−1^ total FA range (Table 2). After a 6-month storage period, a significant increase (*p* < 0.05) was detected in all kinds of samples. A comparison of the batches revealed higher average values in samples corresponding to the OCL-30 glazing system. Differences between the Control and OCL-10 batches were found to be significant (*p* < 0.05) after 6 months of frozen storage.

Concerning the lipid oxidation determination, the PI has shown to afford complementary information on the development of this deteriorative mechanism. Remarkably, it can provide knowledge on the possible variation of the PUFA level (i.e., C20:5ω3 and C22:6ω3 fatty acids) during marine species processing in general and has been found to be directly related to the nutritional value [32,45]. In the present research, the assessment of the PI indicated a slight increase with the frozen storage time, so that the highest average values were observed after 6 months in all kinds of samples (Table 3). A comparison of the batches indicated that the highest average values were obtained in fish corresponding to the OCL-30 batch. Differences between the Control batch were found to be significant (*p* < 0.05) at the end of the experiment.

Values obtained for the PI (Table 3) agree with the trends previously mentioned for the STFA and PUFA values (Table 2), with the frozen storage time and the glazing condition used. According to the preservative effect detected for the PI value, the presence of an aq. solution of OCL in the packing medium led to an increased PI average value in canned mackerel (*S. colias*) [24]. Additionally, higher average PI levels were also detected in canned horse mackerel (*T. trachurus*) that was previously stored under frozen conditions by including an aq. OCL solution in the packing medium [36]. A protective effect on the PI was also observed in frozen Atlantic Chub mackerel (*S. colias*) when an aq. solution of alga *C. stricta* was included in the glazing medium [32].

Concerning the PUFA series (namely, ω3 and ω6), great emphasis has been given to the ω3/ω6 ratio in seafood and food in general [46,47]. To prevent cardiovascular, neurological, and inflammatory disorders, the European Nutritional Society reported that a human diet with a ω3/ω6 ratio of 1/5 or higher would have health benefits [48]. Additionally, the World Health Organization (WHO) currently recommends that this ratio should be higher than 0.1 in the human diet [49]. 

In the current research, healthy ω3/ω6 values were detected in all kinds of samples, being included in all cases in the 14.37–16.58 range (Table 3). Increases of the average value were detected in the OCL-10 (month 6) and OCL-30 (months 3 and 6) batches with the storage time; however, differences were not found significant (*p* > 0.05). Concerning the effect of the OCL presence in the glazing medium, no significant differences (*p* > 0.05) were observed among batches. Remarkably, average values obtained in both OCL-treated batches were higher than those obtained in their corresponding counterparts from the Control condition at the end of the storage period.

According to the current study, no effect on the ω3/ω6 ratio of canned mackerel (*S. colias*) was observed by the presence of OCL in the coating medium [24]. Contrarily, higher average PI levels were detected in frozen mackerel (*S. colias*) by applying a glazing condition that included an aqueous solution of alga *C. stricta* [32].

### 3.4. Determination of the Lipid Hydrolysis Development of Fish Muscle

A strong formation (*p* < 0.05) of FFA could be observed in all kinds of samples after 3 months of frozen storage (Table 1). An additional lipid hydrolysis increase was detected at the end of the experiment. After 3 and 6 months of frozen storage, the samples corresponding to the OCL-30 glazing condition led to lower average values than samples from the Control and OCL-10 batches. However, the differences were not found to be significant (*p* > 005). Therefore, no effect could be concluded for the OCL-glazing treatment on the FFA formation.

The present FFA values can be considered the result of several factors. On the one hand, the freezing, frozen storage, and thawing processes can lead to hydrolysis development of higher-molecular weight lipid classes like phospholipids (PL) and triacylglycerols (TG) by endogenous enzyme (i.e., lipases and phospholipases) activity [30,32,50]. On the other hand, FFA are reported to be more prone to be oxidised or broken down during the freezing, frozen storage, and thawing processes than TG and PL [51]; this behaviour has been explained on the basis of showing a greater accessibility to pro-oxidant molecules. Accordingly, this effect would lead to an FFA value decrease. Finally, the presence of preserving molecules in the OCL may partially inhibit the oxidation development of FFA. According to previous studies on seafood frozen storage [34,52], the first effect has been revealed to be more important in the current study, while no preserving effect could be proved for the presence of OCL in the glazing system.

In agreement with the current research, no effect on the FFA value in canned mackerel (*S. colias*) was obtained by the OCL presence in the packing medium [24]. Contrarily, canned horse mackerel (*T. trachurus*) that was previously subjected to different frozen storage periods showed an increased FFA content by the addition of an OCL solution to the packing medium [36].

The effect on the FFA content by addition of different kinds of natural preservative compounds to the glazing system has been studied previously. Thus, glazing including essential oil (thyme, clove, and sage) extracts has been shown to have an inhibitory effect on the FFA formation in frozen rainbow trout (*O. mykiss*) fillets [8]. An inhibitory effect on the lipid hydrolysis development was also observed by including a plant-derived (i.e., saponin-free quinoa, *C. quinoa*) extract during the frozen storage of mackerel (*S. scombrus*) [42]. Related to marine sources of natural preservative compounds, a glazing system including a rainbow sardine (*Dussumieria acuta*) protein hydrolysate led to a decreased formation of FFA in frozen (−18 °C) black pomfret (*Parastromateus niger*) fillets [53]. A lower FFA value was detected in frozen mackerel (*S. colias*) by including a jumbo squid (*D. gigas*) skin extract in the glazing system [44]. A lower lipid hydrolysis development was found in frozen mackerel (*S. colias*) by applying a glazing condition including an alga *C. stricta* extract [32]. A slight decrease of the FFA content was detected by Naseri et al. [10] in frozen rainbow trout (*O. mykiss*) by using cold-water (1.0 °C for 20 s) glazing. Recently, an inhibitory effect on FFA formation was detected in frozen shrimp (*S. melantho*) if glazed with an aqueous rosemary (*R. officinalis*) extract [9].

## 4. Conclusions

A preservative strategy has been developed based on the employment of a glazing system including an aq. OCL solution. As a result, an inhibitory effect (*p* < 0.05) on lipid oxidation development (formation of TBARS and fluorescent compounds) was detected in frozen fish corresponding to the highest OCL concentration glazing tested. Additionally, a preservative effect on the PUFA (PI assessment) content was proved. Contrarily, no effect on the FFA content and on the ω3/ω6 ratio was detected. An increased lipid stability was identified with the addition of the OCL solution to the glazing system. This effect was explained on the basis of the presence of antioxidant compounds in the cooking liquor resulting from octopus processing.

The present research constitutes a novel and beneficial strategy to increase the quality of frozen fish, which matches with current efforts related to the replacement of synthetic preservative compounds by others obtained from natural sources. Additionally, the fact of employing a waste material, liquor resulting from the octopus processing, is in agreement with international interests in the search for technologies and strategies that increase the circular economy and environmental sustainability. 

Further research would be necessary in order to establish the main components of the current OCL that are responsible for the protection effect on the lipid fraction. Additional oncoming studies would also consist of the optimisation and scale-up of the experimental conditions (i.e., OCL concentration and glazing preparation), and the kind of seafood product to be commercialised (whole pieces, fillets, etc.) when applied to different kinds of marine species, including high-value fatty species such as tuna, bonito, or salmon. Such studies ought to also focus on the physical and sensory properties of the frozen product, in order to assure the consumers’ acceptance of the resulting frozen products. Finally, and based on the remarkable effect of glazing treatment and frozen storage in general, further studies ought to focus on changes produced in the protein fraction.

## Figures and Tables

**Table 1:** Determination of lipid damage * in frozen horse mackerel subjected to different glazing conditions **.

	Frozen Storage Time (Months)	Glazing Condition		
		Control	OCL-10	OCL-30
Peroxide value (meq. active oxygen kg^−1^ lipids)	Initial fresh	0.46 A (0.15)	0.46 A (0.15)	0.46 A (0.15)
	3	1.34 abB (0.55)	0.85 aB (0.11)	1.51 bC (0.13)
	6	0.91 aAB (0.32)	0.82 aB (0.18)	0.96 aB (0.04)
TBARS value (mg malondialdehyde kg^−1^ muscle)	Initial fresh	0.05 A (0.02)	0.05 A (0.02)	0.05 A (0.02)
	3	0.74 bB (0.19)	1.01 bB (0.09)	0.42 aB (0.07)
	6	1.26 bC (0.11)	1.10 abB (0.20)	0.94 aC (0.09)
Fluorescence ratio	Initial fresh	0.52 A (0.04)	0.52 A (0.04)	0.52 A (0.04)
	3	2.02 bB (0.64)	1.09 aB (0.17)	1.01 aB (0.07)
	6	2.25 bB (0.17)	1.25 aB (0.28)	1.35 aB (0.43)
FFA (mg kg^−1^ muscle)	Initial fresh	20.0 A (4.1)	20.0 A (4.1)	20.0 A (4.1)
	3	570.2 aB (87.3)	523.8 aB (64.6)	507.0 aB (65.3)
	6	1357.2 aC (98.3)	1364.0 aC (92.9)	1211.9 aC (85.5)

* Mean values of three (*n* = 3) replicates. Standard deviations are expressed in brackets. For each frozen storage time, lowercase letters (a,b) indicate significant differences (*p* < 0.05) as a result of the glazing condition. For each glazing condition, capital letters (A,B,C) indicate significant differences (*p* < 0.05) as a result of the storage time. ** Glazing conditions: Control (frozen fish with only water as glazing medium), OCL-10 (glazing system including an aq. 10% solution of octopus-cooking liquor), and OCL-30 (glazing system including an aq. 30% solution of octopus-cooking liquor). Abbreviations: TBARS (thiobarbituric acid reactive substances) and FFA (free fatty acids).

**Table 2:** Determination of the fatty acid (FA) group content (g 100 g^−1^ total FA) * in frozen horse mackerel subjected to different glazing conditions **.

FA Group ***	Frozen Storage Time (Months)	Glazing Condition		
		Control	OCL-10	OCL-30
STFA	Initial fresh	33.98 A (0.10)	33.98 A (0.10)	33.98 B (0.10)
	3	34.15 aAB (0.88)	33.64 aA (1.42)	33.28 aB (1.31)
	6	36.06 bB (1.48)	34.62 bA (0.88)	28.52 aA (1.15)
MUFA	Initial fresh	20.77 B (4.26)	20.77 B (4.26)	20.77 B (4.26)
	3	21.16 aB (2.24)	20.46 aB (2.35)	18.90 aB (6.24)
	6	10.18 aA (1.61)	8.99 aA (0.98)	9.23 aA (0.91)
PUFA	Initial fresh	45.25 A (4.36)	45.25 A (4.36)	45.25 A (4.36)
	3	44.89 aA (2.99)	44.90 aA (2.04)	47.83 aA (7.47)
	6	53.77 aB (3.03)	56.39 aB (1.85)	62.24 bB (2.02)
Total ω3 FA	Initial fresh	42.36 A (4.42)	42.36 A (4.42)	42.36 A (4.42)
	3	42.05 aA (3.22)	41.98 aA (1.93)	45.00 aAB (7.44)
	6	50.18 aB (3.50)	53.09 aB (1.92)	58.70 bB (2.12)

* Mean values of three (*n* = 3) replicates. Standard deviations are expressed in brackets. For each frozen storage time, lowercase letters (a,b) indicate significant differences (*p* < 0.05) as a result of the glazing condition. For each glazing condition, capital letters (A,B) indicate significant differences (*p* < 0.05) as a result of the storage time. ** Glazing conditions as expressed in Table 1. *** Abbreviations: STFA (saturated fatty acids), MUFA (monounsaturated fatty acids), and PUFA (polyunsaturated fatty acids).

**Table 3:** Determination of fatty acid ratios * in frozen horse mackerel subjected to different glazing conditions **.

FA Ratio	Frozen Storage Time (Months)	Glazing Condition		
		Control	OCL-10	OCL-30
Polyene index	Initial fresh	1.78 AB (0.16)	1.78 A (0.16)	1.78 A (0.16)
	3	1.80 aA (0.12)	1.78 aA (0.12)	1.92 aAB (0.33)
	6	2.00 aB (0.16)	2.19 abB (0.15)	2.32 bB (0.09)
Total ω3/Total ω6	Initial fresh	14.72 A (1.88)	14.72 A (1.88)	14.72 A (1.88)
	3	14.95 aA (2.40)	14.37 aA (0.79)	15.94 aA (2.64)
	6	14.23 aA (2.66)	16.18 aA (1.62)	16.58 aA (1.14)

* Mean values of three (*n* = 3) replicates. Standard deviations are expressed in brackets. For each frozen storage time, lowercase letters (a,b) indicate significant differences (*p* < 0.05) as a result of the glazing condition. For each glazing condition, capital letters (A,B) indicate significant differences (*p* < 0.05) as a result of the storage time. ** Glazing conditions as expressed in Table 1.

## Data Availability

All related data and methods are presented in this paper. Additional inquiries should be addressed to the corresponding author.

## References

[1] Alizadeh E., Chapleau N., de Lamballerie M., Le-Bail A. (2007). Effect of different freezing processes on the microstructure of Atlantic salmon (*Salmo salar*) fillets. Innov. Food Sci. Emerg. Technol..

[2] Rustad T., Nollet L.M., Toldrá F. (2010). Lipid oxidation. Handbook of Seafood and Seafood Products Analysis.

[3] Özoğul Y., Nollet L.M., Toldrá F. (2010). Methods for freshness quality and deterioration. Handbook of Seafood and Seafood Products Analysis.

[4] Vanhaecke L., Verbeke W., De Brabander H. (2010). Glazing of frozen fish: Analytical and economical challenges. Anal. Chim. Acta.

[5] Yerlikaya P., Gokoglu N. (2010). Inhibition effects of green tea and grape seed extracts on lipid oxidation in bonito fillets during frozen storage. Int. J. Food Sci. Technol..

[6] Tan M., Li P., Yu W., Wang J., Xie J. (2019). Effects of glazing with preservatives on the quality changes of squid during frozen storage. Appl. Sci..

[7] Wang J., Yu W., Xie J. (2020). Effect of glazing with different materials on the quality of tuna during frozen storage. Foods.

[8] Çoban O. (2013). Evaluation of essential oils as a glazing material for frozen rainbow trout (*Oncorhynchus mykiss*) fillet. J. Food Proc. Preserv..

[9] Shi J., Lei Y., Shen H., Hong H., Yu X., Zhu B., Luo Y. (2019). Effect of glazing and rosemary (*Rosmarinus officinalis*) extract on preservation of mud shrimp (*Solenocera melantho*) during frozen storage. Food Chem..

[10] Naseri M., Abedi E., Vafa S., Torri L. (2020). Comparative investigation of physico-chemical and sensory properties of glazed and non-glazed frozen rainbow trout (*Oncorhynchus mykiss*) thawed with different methods by principal component analysis. Iran Agric. Res..

[11] Wang X.S., Xie J. (2021). Quality attributes of horse mackerel (*Trachurus japonicus*) during frozen storage as affected by double-glazing combined with theaflavins. Int. J. Food Prop..

[12] Gonçalves A., Gindri C. (2009). The effect of glaze uptake on storage quality of frozen shrimp. J. Food Eng..

[13] Soares N., Mendes T., Vicente A. (2013). Effect of chitosan-based solutions applied as edible coatings and water glazing on frozen salmon preservation—A pilot-scale study. J. Food Eng..

[14] Blanco M., Sotelo C.G., Chapela M.J., Pérez-Martín R.I. (2007). Towards sustainable and efficient use of fishery resources: Present and future trends. Trends Food Sci. Technol..

[15] Venugopal V., Venugopal V. (2009). Marine product for health care. Marine Product for Health Care.

[16] Shahidi F. (2007). Maximising the Value of Marine By-Products.

[17] Arvanitoyannis I.S., Kassaveti A. (2008). Fish industry waste: Treatments, environmental impacts, current and potential uses. Int. J. Food Sci. Technol..

[18] Rustad T., Storro I., Slizyte R. (2011). Possibilities for the utilisation of marine by-products. Int. J. Food Sci. Technol..

[19] Atef M., Ojagh M. (2017). Health benefits and food applications of bioactive compounds from fish byproducts: A review. J. Funct. Foods.

[20] Lee S.Y., Hur S.J. (2017). Antihypertensive peptides from animal products, marine organisms, and plants. Food Chem..

[21] Lin Y., Tang X., Xua L., Wanga S. (2019). Antibacterial properties and possible action mechanism of chelating peptides-zinc nanocomposite against *Escherichia coli*. Food Cont..

[22] Oh H.S., Kang K.T., Kim H.S., Lee J.H., Jee S.J., Ha J.H., Kim J.J., Heu M.S. (2007). Food component characteristics of sea-food cooking drips. J. Korean Soc. Food Sci. Nutr..

[23] Kim Y.J., Kim H.J., Choi J.I., Kim J.H., Chun B.S., Ahn D.H., Kwon J.H., Kim Y.J., Byun M.W., Lee J.W. (2008). Effect of electron beam irradiation on the physiological activities of cooking drips from *Enteroctopus dofleini*. J. Korean Soc. Food Sci. Nutr..

[24] Malga J.M., Trigo M., Martínez B., Aubourg S.P. (2022). Preservative effect on canned mackerel (*Scomber colias*) lipids by addition of octopus (*Octopus vulgaris*) cooking liquor in the packaging medium. Molecules.

[25] AOAC (1995). Official Methods of Analysis of Association of Official Analytical Chemists International.

[26] Bligh E., Dyer W. (1959). A rapid method of total lipid extraction and purification. Canadian J. Biochem. Physiol..

[27] Herbes S.E., Allen C.P. (1983). Lipid quantification of freshwater invertebrates: Method modification for microquantitation. Canadian J. Fish. Aq. Sci..

[28] Chapman R., McKay J. (1949). The estimation of peroxides in fats and oils by the ferric thiocyanate method. J. Am. Oil Chem. Soc..

[29] Vyncke W. (1970). Direct determination of the thiobarbituric acid value in trichloracetic acid extracts of fish as a measure of oxidative rancidity. Fette Seifen Anstrichm..

[30] Aubourg S.P., Piñeiro C., González M.J. (2004). Quality loss related to rancidity development during frozen storage of horse mackerel (*Trachurus trachurus*). J. Am. Oil Chem. Soc..

[31] Lowry R., Tinsley I. (1976). Rapid colorimetric determination of free fatty acids. J. Am. Oil Chem. Soc..

[32] Oucif H., Ali Mehidi S., Aubourg S.P., Abi-Ayad S.M. (2020). Antioxidant effect of an aqueous extract of alga *Cystoseira stricta* during the frozen storage of Atlantic Chub mackerel (*Scomber colias*). Bulgarian Chem. Com..

[33] Ahn C.B., Kim H.R. (1996). Processing of the extract powder using skipjack cooking juice and its taste compounds. Korean J. Food Sci. Technol..

[34] Romotowska P., Karlsdóttir M., Gudjunsdóttir M., Kristinsson H., Arason S. (2016). Influence of feeding state and frozen storage temperature on the lipid stability of Atlantic mackerel (*Scomber scombrus*). Int. J. Food Sci. Technol..

[35] Tironi V., Tomás M., Añón M.C. (2002). Structural and functional changes in myofibrillar proteins of sea salmon (*Pseudopercis semifasciata*) by interaction with malondialdehyde (RI). J. Food Sci..

[36] Méndez L., Trigo M., Zhang B., Aubourg S.P. (2022). Antioxidant effect of octopus by-products in canned horse mackerel (*Trachurus trachurus*) previously subjected to different frozen storage times. Antioxidants.

[37] Choi J.I., Kim Y.J., Sung N.Y., Kim J.H., Ahn D.H., Chun B.S., Cho K.Y., Byun M.W., Lee J.W. (2009). Investigation on the increase of antioxidant activity of cooking drip from *Enteroctopus dofleini* by irradiation. J. Korean Soc. Food Sci. Nutr..

[38] Jao C.L., Ko W.C. (2002). 1,1-Diphenyl-2-picrylhydrazyl (DPPH) radical scavenging by protein hydrolyzates from tuna cooking juice. Fish. Sci..

[39] Cheong H.S. (2007). Antioxidant effect of histidine containing low molecular weight peptide isolated from skipjack boiled extract. Korean J. Food Cook. Sci..

[40] Oliveira D., Bernardi D., Drummond F., Dieterich F., Boscolo W., Leivas C., Kiatkoski E., Waszczynskyj N. (2017). Potential use of tuna (*Thunnus albacares*) by-product: Production of antioxidant peptides and recovery of unsaturated fatty acids from tuna head. Int. J. Food Eng..

[41] Li K.X., Ding H.P., Zhou X.J., Zhang J., Zhang H.Q., Liu L.P. (2020). Antioxidant and deoridizing treatment of tuna steamed juice and development of its flavor salad. Sci. Technol. Food Ind..

[42] Trigo M., Rodríguez A., Dovale G., Pastén A., Vega-Gálvez A., Aubourg S.P. (2019). The effect of glazing based on saponin-free quinoa (*Chenopodium quinoa* Willd.) extract on the lipid quality of frozen fatty fish. LWT-Food Sci. Technol..

[43] Cavonius L., Undeland I. (2017). Glazing herring (*Clupea harengus*) fillets with herring muscle press juice: Effect on lipid oxidation development during frozen storage. Int. J. Food Sci. Technol..

[44] Ezquerra-Brauer J.M., Trigo M., Torres-Arreola W., Aubourg S.P. (2018). Preservative effect of jumbo squid (*Dosidicus gigas*) skin extract as glazing material during the frozen storage of Atlantic Chub mackerel (*Scomber colias*). Bulgarian Chem. Com..

[45] Aubourg S.P., Trigo M., Prego R., Cobelo-García A., Medina I. (2021). Nutritional and healthy value of chemical constituents obtained from Patagonian squid (*Doryteuthis gahi*) by-products captured at different seasons. Foods.

[46] Uauy R., Valenzuela A. (2000). Marine oils: The health benefits of n-3 fatty acids. Nutrition.

[47] Komprda T. (2012). Eicosapentaenoic and docosahexaenoic acids as inflammation-modulating and lipid homeostasis influencing nutraceuticals: A review. J. Funct. Foods.

[48] Simopoulos A.P. (2002). The importance of the ratio of omega-6/omega-3 essential fatty acids. Biomed. Pharm..

[49] Kumari P., Kumar M., Reddy C.R., Jha B., Domínguez H. (2013). Algal lipids, fatty acids and sterols. Functional Ingredients from Algae for Foods and Nutraceuticals.

[50] Aubourg S.P., Rey-Mansilla M., Sotelo C. (1999). Differential lipid damage in various muscle zones of frozen hake (*Merluccius merluccius*). Z. Lebensm. Unters. Forsch..

[51] Aubourg S.P. (2001). Fluorescence study of the prooxidant activity of free fatty acids on marine lipids. J. Sci. Food Agric..

[52] Losada V., Barros-Velázquez J., Aubourg S.P. (2007). Rancidity development in frozen pelagic fish: Influence of slurry ice as preliminary chilling treatment. LWT-Food Sci. Technol..

[53] Taheri A. (2015). Antioxidative effect of rainbow sardine (*Dussumieria acuta*) protein hydrolysate on lipid and protein oxidation in black pomfret (*Parastromateus niger*) fillet by glazing. J. Aq. Food Prod. Technol..

